# Empowering AI assisted clinical drug development: tactics to address data bias, the digital divide and missing patient populations through AI and digital solutions

**DOI:** 10.3389/fdgth.2026.1880641

**Published:** 2026-08-12

**Authors:** Dimitris Papanicolaou, Sotirios Perdikeas, Graham B. Jones

**Affiliations:** 1 Clinical Innovation, Novartis Pharmaceuticals, East Hanover, NJ, United States; 2 Global Clinical Operations, Novartis AG, Basel, Switzerland; 3 Clinical Innovation, Novartis Pharmaceuticals, Cambridge, MA, United States; 4 Clinical and Translational Science Institute, Tufts University Medical Center, Boston, MA, United States

**Keywords:** AI, data bias, engagement, health data, heterogeneity, microtargeting, sub-populations

## Abstract

The use of AI methodologies in drug development holds great potential to expedite clinical trials by helping to identify patients most likely to respond favorably to a given medication. To fully harness the benefits of such algorithmic approaches to precision medicine however requires that the datasets from which such predictive analyses are performed are fully representative of the populations intended to benefit. Due to a multitude of factors, there remains extant need to increase the heterogeneity of these data including the contribution of under-represented and other absent populations. There is also a parallel need to broaden patient representation in the clinical trials themselves which are used to demonstrate efficacy of predictive models deployed. Herein we outline tactics, approaches and measures that could be deployed to drive these elements, the potential impact of such on precision medicine, and the ethical, legal, and privacy issues which need to be considered. The benefits of such would be myriad, including helping realize more fully the potential of precision medicine, which aims to pair the most appropriate care and medications with patients based on their individual phenotypic and pharmacogenomic profiles.

## Introduction

One of the first recorded efforts to understand the systematic origins of disease is attributed to the Greek philosopher Alcmaeon of Croton approximately 500 B.C.E. His early efforts included theories on the contribution of environmental, nutritional and lifestyle factors to illness (1). Some 2,500 years later our understanding of causal factors and systems biology owe much to the diligence and application of such applied scientific method. Among striking recent advances is the deployment of machine learning, large language processing and deep learning techniques to analyze and predict patient response rates from aggregated health datasets (2, 3). These AI methods have allowed more effective and efficient trial design through tailored cohort selection and heightened precision in identifying response signals (4). Several large data sets have been assembled and are deployed by the rapidly evolving bioinformatics industry supporting the clinical drug development community (5). A noted limitation however is that the composition of these data sets is biased to certain patient populations whose data was used to assemble (6). The consequence of such is that the data may not be fully representative of populations who may enroll in clinical trials, limiting the effectiveness of predictive models derived from the primary data (7). Additionally, there exist perennial barriers to achieving broad patient representation in contemporary clinical trials, despite concerted efforts to encourage (8, 9). Herein we examine the origins of these biases and propose methods to improve representation and engagement, essential elements if we are to fully harness the power of AI methodologies in clinical development. We first (i) address need by reviewing clinical outcomes and levels of patient engagement among sub-populations alongside differences based on genetic, socioeconomic and environmental factors. We then (ii) examine potential approaches for patients to share data and participate in trials, the use of digital and AI driven micro-targeting methods to refine trial design and discuss the impact of fully representative datasets. Finally (iii) we outline potential paths forward and examine caveats to be mindful of in implementation. Manuscripts highlighted represent a cross section of recent literature derived from the medical, clinical, scientific, social science and technology fields and are intended to guide the reader to pursue further analysis.

## Clinical outcomes among sub-populations

**Figure 1:**
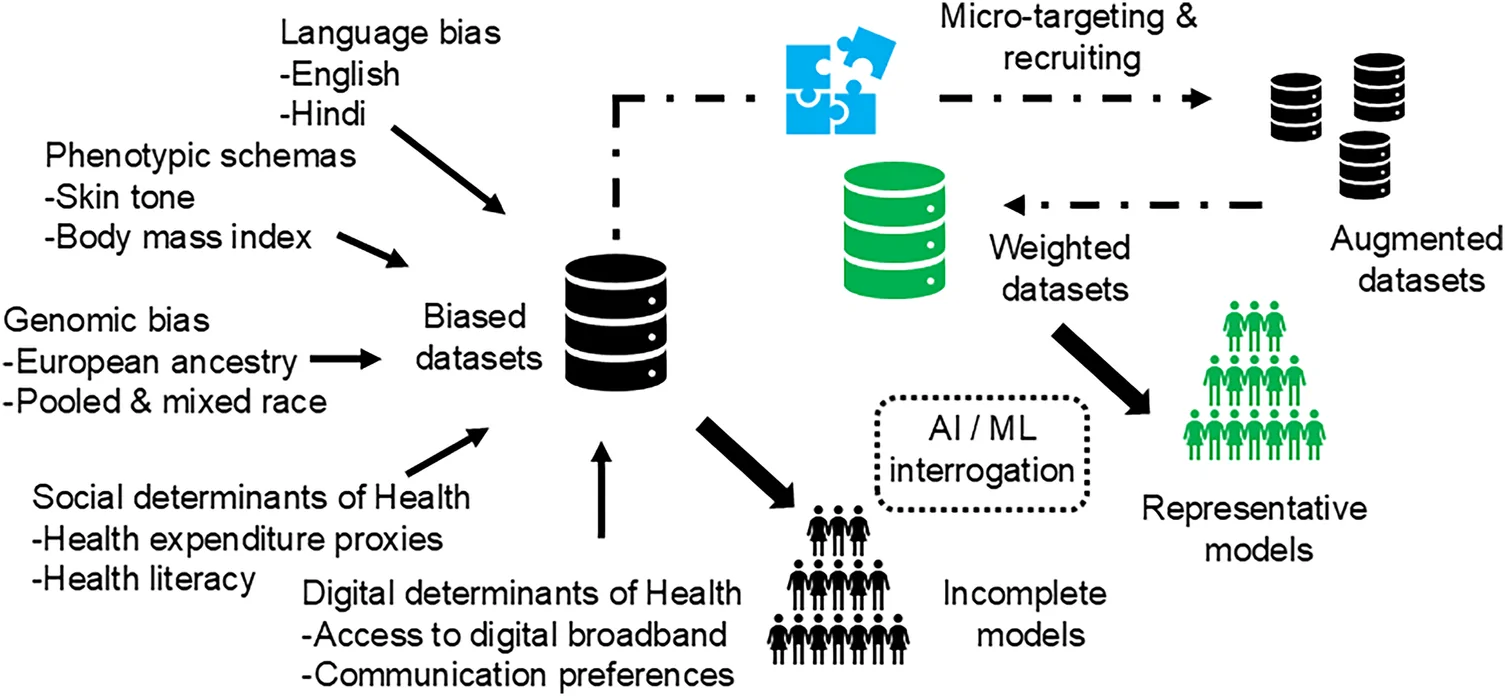
AI augmentation to address diversity biases stemming from missing data.

The application of AI technologies in clinical trial design has rapidly emerged as a powerful enabling tool. Supporting this ever increasing field are numerous deeply populated databases composed of data amassed from electronic health records, clinical outcomes, real world evidence and synthetic control arms. This said, it is acknowledged that biases and other limitations restrict the power and generalizability of the data stemming from a general lack of heterogeneity of the patient data used to populate. As examples, a large proportion of genomic data is derived from persons of European ancestry whereas Hispanic, Asian, African and mixed race individuals are under-represented (Figure 1) (10–12). In the case of patient generated written data the models over emphasize certain languages such as English and Hindi simply based on data availability contributing to language induced bias (Figure 1) (13). Other biases may relate to phenotypic attributes such as skin-tone (14), and unintentional biases may also stem from the subjects' level of access to health care itself and the so-called digital divide which impacts telehealth services, the sharing of electronic health data, and the likelihood of trial enrollment through online digital patient portals (Figure 1) (15–17). Collectively these data biases can lead to generation of incomplete models when subjected to AI based analysis, and it is generally acknowledged that augmentation of the datasets is needed in order to have full confidence that representative modelling can be performed and we reduce vulnerability to so called “pernicious bias” (Figure 1) (18–20). Among myriad factors, another potential source of missing data stems from patients simply seeking care from multiple providers who are on different networks (21).

## Differences based on genetic, socioeconomic and environmental factors

The criticality of fully representative datasets can be easily understood when one considers the impact of race and ethnicity on health and clinical outcomes. For example, Ashkenazi Jewish persons have increased susceptibility to a number of genetic diseases including cystic fibrosis, Gaucher disease, mucolipidosis, and Niemann-Pick disease (22). Globally, such traits are often referred to as founder effects, when groups of people with particular genetic predispositions migrate and establish new populations (23). In the area of drug metabolism, known differences exist between Caucasians and Asians in CYP450 enzyme activity particularly CYP2D6 and CYP2C19 (24), Han Chinese show differences in CYP2C19, CYP2C9, and CYP3A5 (25), and Africans, African Americans and Amerindians show differences in CYP2D6*10 alleles (26). Additionally, when considering migrant populations the use of broad grouping descriptors such as “Asian American” or “Latino/a” can lead to inaccurate assumptions based on subtle differences within the groups (27, 28). Health differences between populations among neighboring states in Europe have been examined and contrasts noted among numerous measures including antibiotic resistance, infectious disease rates, and cancer survival. In addition to genomics, these outcomes can reflect differences in health care availability, environmental factors such as air pollution and water quality, lifestyle factors of the populations (e.g., alcohol consumption, dietary composition) and even factors relating to differences in religious doctrine (29). Genome analysis is also insightful to provide insight to ancestral and migratory patterns of populations over time, exemplified by studies from central Europe, where genetic variations among western Balkan populations support the theory that the peninsula was a major route for gene flows from the middle east through to Europe (30). Similarities between groups have been defined using haplotype analysis, suggesting they share a common paternal ancestry derived from pre-ottoman times (31). Migratory actions can as expected also lead to anomalies among new populations. Results from a large Australian study indicated that following migration to Western countries, the health status of Chinese immigrants worsened over time with the increasing risk of developing heart disease, obesity, and diabetes (32). Similar studies have been performed in the USA revealing insight to disease dynamics and outcomes related to integration in new nation states (33). Disease hotspots can also emerge. For example in the case of nasopharangeal cancer, two of the highest prevalences are found in Inuit Canada and Guandong province in China. Possible environmental factors have been identified, including methods for food preparation involving salting of seafood (34). Collectively, the potential for such health outcomes disparities to be masked through aggregated data used for AI model generation poses a substantial risk for incomplete and biased predictions.

## Levels of patient engagement vary across sub-populations

**Figure 2:**
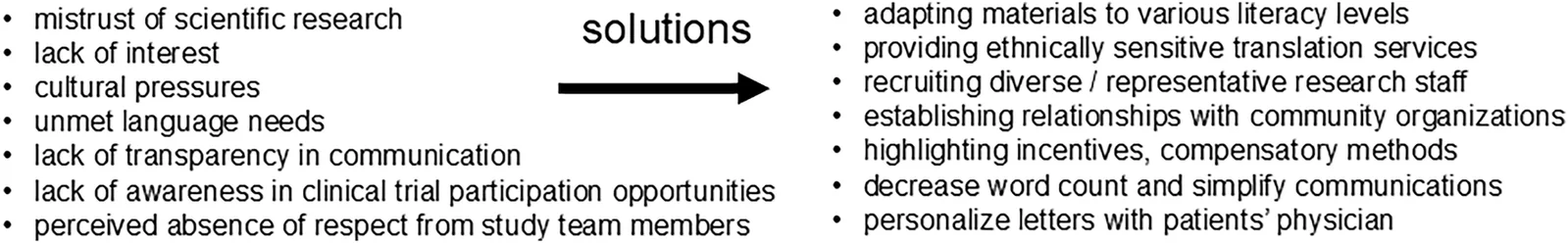
Origins of disengagement in healthcare and potential solutions.

Another complicating factor in the quest for representative data relates to differences in levels of health engagement between populations. People from different backgrounds have considerable variation in the level of trust with managed healthcare, and especially their appetite for sharing health data through electronic records (35–37). These differences also surface when attempting to recruit patients into clinical trials, where considerable challenges are encountered establishing fully representative cohorts (9, 38). There exist sometimes stark contrasts between populations with regard to belief in the actual value of health services, and this could be a source of error in aggregated population level data (Figure 2). For example, major differences in attitudes towards mental health care have been noted between sub-populations in Europe (39). There is also the consideration of engagement mode, particularly among digital channels. Studies reveal lowered appetite for smartphone based engagement versus text based approaches among certain populations when engaging with HCP's (40), but high appetite for community based mHealth apps in situations involving disease management (36), Additionally, it has been noted that patient feedback captured in the form of tweets may under represent contributions from subjects who communicate using specific dialects and vernacular within languages (41). Nonetheless, it is noted that the high levels of smartphone use among ethnic and racial minorities represents an ideal opportunity for engagement using digital technology, and has been demonstrated to good effect in studies on follow up care in cervical cancer (42).

## How might we encourage the missing patients to share data and participate in trials?

Concerted efforts will be required in order to close the evident data gap and likewise broaden participation in managed healthcare and in clinical trials. In this regard there is likely no universal solution and multiple channels will need to be explored as we describe herein. At a minimum this will require careful and sustained effort in sociocultural messaging (Figure 2) (9). For communications, both the language and visual elements of any materials intended to capture patient intertest need to be tailored to echo cultural and community norms of the target populations (Figure 2) (9). In some populations this for example requires not only translation of materials but also adaptation to resonate with the audience. Highly targeted, direct, and culturally adapted communications can help establish rapport and foster trust and psychological safety, which can then translate to improved engagement (Figure 2). Herein we outline some tactics and strategies which might be capitalized upon to explore these value added elements:

### Engaging through institutions

Institutions through which people share affinity with are an obvious locus from which to drive health engagement. Moreover, the ability to communicate and develop digital and AI technologies at scale through institutional adoption represents an attractive possibility. Some options are more digitally adaptable than others, but most offer potential. Among these, health awareness campaigns and recruiting drives have shown some success in hospital and long term care facilities, both for patients, their relatives and visitors (43). Likewise military establishments have shown promise, with the US even home to its own hospital network the Veterans Administration (44). Prisons are another venue where heightened interest in health related matters among inmates has been noted (45). In an effort to attract younger demographics colleges and universities are a logical option, and the younger generation has demonstrable appetite and familiarity with digital records and service provision (46). More broadly, large employers have potential to engage with their workforce, and it is noteworthy that a number of behavioral health services are now provided through the employment base (47). In terms of linking to daily routines, faith based organizations (churches, fellowships and religious organizations) offer a potentially ideal venue, skewed in many countries to older demographics (48). It is also noteworthy that a number of communities globally rely heavily on advice from pastors or their equivalent for health information, representing a potentially unique portal based on trust (49). There are other options which could benefit from transient captive audiences such as on cruise ships which cater to health conscious (50), those attending outpatient clinics (51), or even when applicants renew driving licenses and are given the option to enroll as organ transplant donors (52). In most of the above examples, digitization of communication channels and record keeping has become commonplace, providing opportunity for AI inspired highly personalized interactions.

### Tie-ins to current events

Health related current events are an ideal opportunity to raise awareness on the importance and value of engagement with the health system. The recent COVID-19 pandemic was a prime example (53), as was the SARS pandemic (54), and the HIV/AIDS epidemic in the 1990′s (55). Natural disasters also often prompt the need to engage in blood drives for the afflicted populations, and there is general understanding on the role of universal donors, universal recipients, and the scarcity of certain blood types viz. AB negative (56). Seasonally, the influenza vaccine campaigns offer an opportunity for more broad engagement around health (57), as do required vaccinations prior to overseas travel to certain destinations (58). A less examined possibility could be to dovetail through associative networks (e.g., political movements, fan groups) where specific groups of people identify through support of defined causes or ideologies and could provide an inroad to promote health related topics (59).

### Launching awareness campaigns

There are myriad channels for the launch of awareness campaigns, and digital versions have largely supplanted traditional print and television media. The ability to micro-target sub-groups with precision using email and text messaging has been used to effect in political campaigns, fueled by AI methods developed by providers in the FAMGA companies and the online retail industry (vide infra) (60). Communicating the importance of health, data, and inclusivity can all benefit from educational content, so community organizations, schools, and organized sport leagues provide good options. Programming which is grounded in case studies, cause and effect on health impacts, and the tenets of patient advocacy “nothing about us without us if for us” resonates well as does the requirement for outcomes research studies to involve patients as stakeholders (61). It has also been noted that conveying to clinical trial participants that they are playing a fundamental role in a process “bigger than the individual self” correlates to increased retention rates (62). At the sub-population level, the role of genetic counselling could be used as a pivot, ranging from genetic diseases to differences in pharmacogenomics based on race, ethnicity and gender, particularly relevant in highly diverse environments (63).

### Identifying new methods for incentivization

The appropriate compensatory mechanisms to engage subjects in clinical trials is oft debated as its ethical considerations are nuanced. Nonetheless, where data gaps are sizeable, financial reward is most routinely used (64). For engagement which may be longer term however, particularly involving EHR's and potentially genomic information, other arrangements may be viable. As witnessed in the college sporting arena in the USA, the Name Image and Likeness (NIL) of athletes has now become monetized, and legal frameworks established on marketing use (65). Could a similar option allow subjects with unique or rare genomic profiles to benefit from such arrangements? One could envisage tie-in with direct to consumer genomic profiling companies, those on bone marrow registries, or those with unique microbiota populations (66). Beyond financial benefit, certain individuals may resonate with the prospect of contributing to modern day medicine. Though the story of Henrietta Lacks is fraught with controversy, her legacy lives on as the HeLa cells are used worldwide in medical research (67). Other incentives could be targeted to specific groups, for example offering health benefits for migrant workers, linking to health seminars at vacation resorts, offering free genetic or microbiome profiling or enrolment in networks that alert users to the availability of new trials or approved medications.

### Leveraging federal level incentives and mandates

One of the established approaches to increasing representation in clinical trials has been legislation and directives from the regulatory bodies including FDA mandates on gender, pediatric populations and ethnicity (68). There are also incentives which encourage drug developers to target specific phenotypes, e.g., expedited approvals pathways in the case of rare and orphan diseases (69). Such incentives could be applied more broadly, e.g., rewarding efforts to include more heterogenous populations in studies and data sharing fora. This could also be applied to companies populating databases, by issuing licenses for those who exceed targets in a similar manner to housing permitting and approvals under community based FHA guidelines (70). There are also incentives provided by funding agencies such as the NIH requirements for data inclusivity and data sharing plans, which must be adhered to by all applicants and form part of the peer review process (71). Another possible touch point at federal level could be in the border control, passporting and immigration services administration. For example, visitors, returners and diaspora could be offered the opportunity to contribute to the national research priorities through health data sharing, which might resonate with subjects' pride in citizenship and patriotism at the point of service (72).

## The next frontier: deploying digital and AI driven micro-targeting methods

For all of the above it would be desirable to be able to develop awareness and recruiting campaigns which are highly personalized in terms of cultural, ethnic and lifestyle content. Such methods have been deployed with some success in commercial advertising and marketing strategies (73, 74), in recent election campaigns (75, 76), and through use of new AI technologies such as the large language model GPT-4 (77). For example, deploying style switching tactics in messaging including language and local dialect can have impact in sub-communities as evidenced by use of French (78), Gaelic (79), or Spanish language in certain populations (80). This messaging could be combined with health related information relevant to specific groups, e.g., risk for genetic disorders, inherited cardiovascular risk factors, or oncologic risk when promoting community based serological screening. The style and format of digital channels is also important to consider, with some ethnic groups expressing clear preferences for particular modalities (81). Herein we outline specific tactics and means to tailor for deployment in healthcare inspired by recent advances (73).

## Examples of successful microtargeting methods

**Figure 3:**
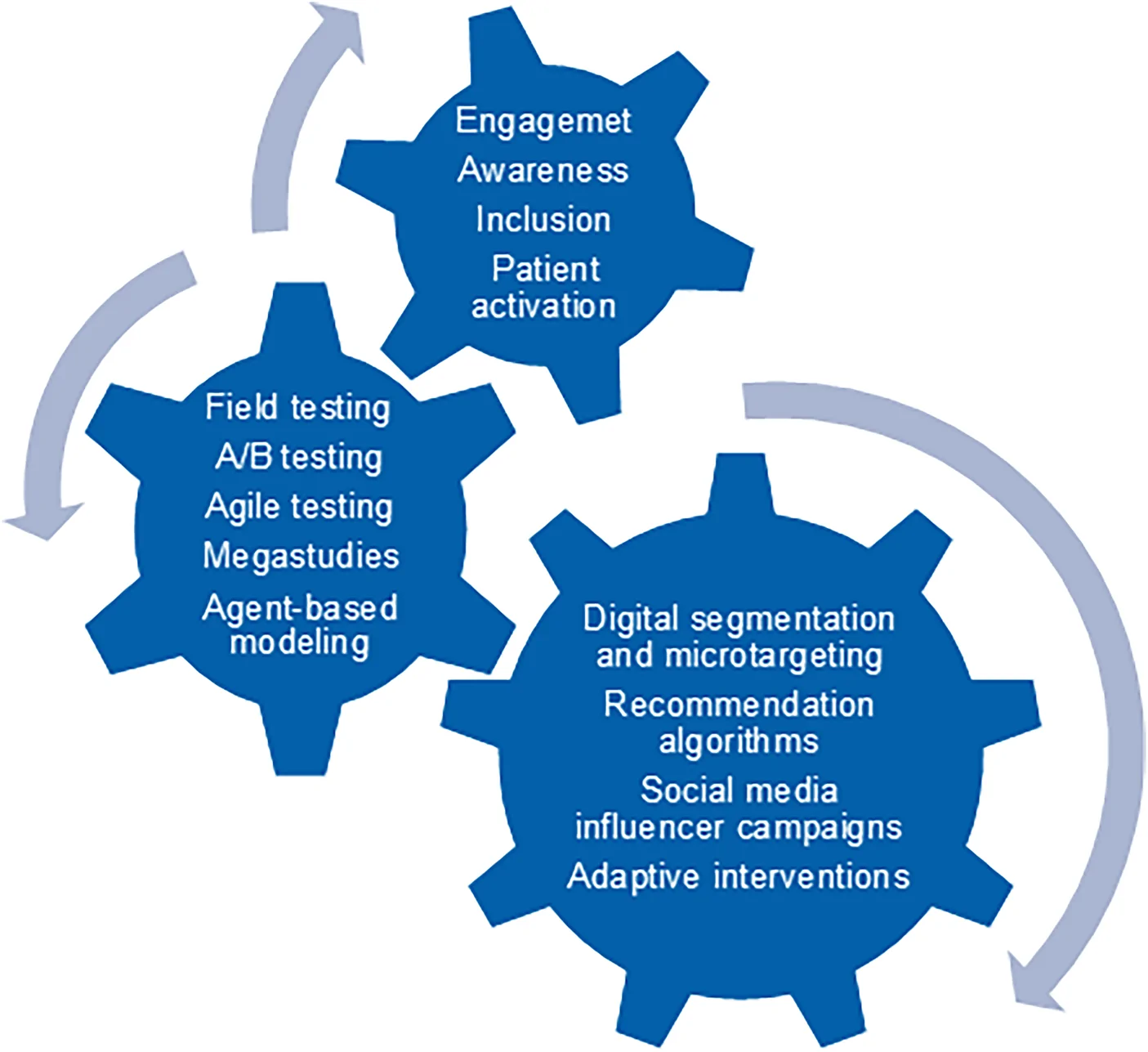
Digital and AI inspired tactics for microtargeting.

Microtargeting approaches often employ market segmentation tactics, involving the identification of discrete individual groups who display distinct characteristics or behaviors and have distinct needs that may resonate with specific communication modalities and messaging tailored to their preferences (Figure 3). Examples include video messaging campaigns directed at persons at risk from skin cancer from Facebook advertisements in states with high rates of tanning bed use (82). Another example involved microtargeting communities with high COVID-19 mortalities through Facebook messaging according to zip codes, which achieved marked reach to majority Black and Latinx populations (83). Web searching tools can also be used to identify sub-populations and have been applied to both Google Ads and Facebook (84).

Another strategy is through social media influencer campaigns, which can be based on large campaigns or through use of targeted microinfluencers (Figure 3). Examples include use in promoting influenza vaccinations among sub-populations, with high rates of engagement observed when native language options could be selected (85). Influencer backed campaigns were also deployed in New York City to promote COVID-19 testing among the LGBTQ + and racial/ethnic minority populations (86).

An approach commonly deployed by video streaming services such as Netflix and Amazon are use of recommendation algorithms which predict individuals reactions based on learned behaviors (Figure 3). Successful application in health care influencing includes public service announcements on smoking cessation where the recommender system was able to target populations, who were subsequently significantly more likely to report subsequent intervention and ultimately cessation (87).

Another approach is the use of just-in-time adaptive interventions delivered by smartphones and digital devices, based on contextual and behavioral data (Figure 3). Examples include interventions to modulate sodium intake among hypertension patients by using geofencing tactics, e.g., when subjects are located near grocery stores or restaurants with just-in-time push notifications (88). One noted caveat is that some 15% of Americans do not own or use a smartphone and this may be higher in certain sub-populations.

Equally important to the four targeting methods described above, are the mechanisms used to refine and ultimately deploy them. The use of A/B testing methods, where messages are refined through platforms such as Facebook, TiKTok, Twitter, and Amazon MTurk, can identify optimized messages for microtargeting (Figure 3). Examples include graphic images used to promote HPV vaccinations among minorities (89), and clinical decision support related to tobacco cessation counselling (90). Other mechanisms used to optimize tailored messages include field testing of concepts such as perceived argument strength (PAS), perceived message effectiveness (PME), and ecological momentary assessment (EMA) (91). These tactics aim to also reduce memory based errors and fatigue among survey participants resulting in more reliable data, and when deployed using mobile devices allow data to be gathered by users in natural environments (91). In many situations the need to evolve microtargeted messages very rapidly becomes a top priority, and agile approaches have been described, including rapid-cycle 4 step models refined during the COVID-19 pandemic (92). There are often differences exposed between intervention ideas measured in different time periods and with different strategies which hinders solution development. One approach to overcome this is the use of so-called megastudies, where numerous interventions are synchronously tested among populations (Figure 3). This was successfully demonstrated among 690,000 consumers to identify best text based nudges to encourage vaccinations (93). In efforts to refine models used to predict behaviors, agent-based modeling is sometimes deployed, wherein concepts (agents) with specific attributes are tested through rule-based encoded mechanisms that relate to behaviors. These simulated approaches have been used to predict attitudes to epidemics based on proximity and other factors (94). Taken together, these approaches might ultimately drive increased awareness, and thus activation of more representative groups of patients (Figure 3). As always however, digital communication modes need to evolve according to consumer proclivities e.g., the increasing trend to engage through virtual reality advancements and AI voice assistants on smartphones.

## Refining clinical trial design

In concert with efforts to broaden participation and engagement using digital and AI inspired approaches there is merit to continually revisiting design of clinical trials themselves with the goal of reducing any real or perceived barriers to patient participation. Social media listening approaches can be used to extract insights regarding the preferences and experience of patients and physicians being part of certain trials. Such insights can be leveraged to design more patient centric protocols and help identify and reduce any unnecessary complexities. For example NLP based analysis of patient forums, advocacy group communications, and public health narratives could be used to help integrate community sentiment and lived experience into feasibility and design decisions. Such approaches have been deployed successfully in specific indications including type 2 diabetes (95) and form the basis of an ongoing long term study by a public-private consortium (96). Assessing patient burden as a function of protocol design is an active field (40) and the potential to develop AI-inspired patient burden indices (e.g., socioeconomic proxies and geography) and representativeness gap scores (e.g., based on age, sex, ethnicity, comorbidity) to flag criteria that disproportionately exclude underrepresented groups is high. Similarly, post-trial AI analysis can be deployed to help close loops between trial designs and actual outcomes, as exemplified by the READI initiative in Europe which involves collaboration with regulators and policymakers (97, 98). Such equity feedback loops can serve to provide key inputs for subsequent trials and help proactively understand whether some sites are overly burdened for example because they try to engage and onboard certain sub populations with unique needs. More broadly, we might rethink how to better enhance our screening and recruitment practices by leveraging insights produced by ePRO analyses, including signals raised through data derived from wearables, apps and telemedicine approaches. By combining region and country based digital access indices, device ownership data, historical dropout patterns and risks it might be possible to generate a digital inclusion risk score per protocol and per country which would be useful for trial design. Ultimately, real world data and registries which are capable of simulating who is likely excluded before the first patient is actually recruited will offer proactive prompts to help enhance study designs.

## The impact of fully representative datasets

The impact of having representative databases will be substantial, allowing AI models to generate more accurate predictions which will lead to faster, more effective clinical trials. Having a more engaged representative population to draw from will allow those trials to be more reflective, realizing the fuller potential of precision medicine, which aims to pair the most appropriate medications with patients based on their pharmacogenomic profile. In addition to developing these drug pairings more efficiently, such a reality would also allow better population level responses to epidemics and pandemics and seasonal challenges such as influenza. It could also expand the utility of much needed repositories used for targeted interventions, including donor registries for rare diseases which match serological or bone marrow profiles. In order to help catalyze this drive towards inclusiveness, concrete steps will be needed across multiple dimensions including health policy, health economics, social messaging, information security and data privacy. Herein we outline some specific steps which could be taken.

## The path forward

Development of comprehensive and fully diversified health data capabilities naturally requires careful consideration of relevant security and privacy measures. Many populations have an innate skepticism of federated approaches to personal health information (where aggregated data are captured and analyzed) (99), and these would need to be addressed cleanly and transparently, with future proofing an essential component. One can imagine advances in secure data exchange being applied to healthcare in the same manner that advances in blockchain technologies helped revolutionize financial transactions used in the crypto currency industry (100). On the global scale the digitization of aspects of our health may open up new possibilities relating to our identities. For example, digitized genomic security markers could become components in next generation passports and provide critical information for travelers, and the countries they visit in times of health crisis or disease outbreak. It is noteworthy that the Twitter archive in the US National Library of Congress currently holds over 170 billion stored personal messages. It seems logical to consider if aspects of our health information, the basis of life itself, might benefit from such curated archival measures. While these of course are longer term questions, there are some concrete next steps that we could pursue in order to expedite the diversification of health datasets and the engagement of the under-represented patients. They include:

1. Promoting transparency regarding the levels of homo/heterogeneity associated with large, aggregated databases offered by commercial vendors through publication of peer-reviewed manuscripts (101–104).
2. Dissemination on the consequences of using non-inclusive data sets in predictive modeling through a call-to-action among premier medical journals and professional societies (105).
3. Highlighting both desired patient recruitment targets and achieved goals for entries published in clinicaltrials.gov.
4. Establishing performance incentives for exceeding inclusion goals in clinical trial recruitments e.g., providing expedited review tokens for registrational trials (106).
5. Introducing measures for state funded healthcare providers which link patient inclusion levels with overall performance criteria.
6. Developing additional guidelines to health advertising standards which promote outreach and resonance with representative patient groups through adaptive messaging techniques (107).
7. Introducing health engagement and awareness nudges in federal document submissions processes including passport, driving license applications.
8. Modifying census bureau documents (or their equivalents) to track health care participation rates and identify service gaps among communities.
9. Establishing dedicated working groups and public-private partnerships on the use of AI and messaging channels to broaden healthcare engagement e.g., IHI in Europe (108).
10. Inviting social media platform owners to increase health advocacy awareness by providing dedicated channel space to influencers, survivors, and patient advocacy organizations.

Many of these measures will take concerted effort over sustained periods, and will face different barriers depending on states, regions and communities. However, there are some that might be advanced more rapidly, for example bullets 1–3 could benefit from active debate among scientific and medical journal editors and their advisory boards. Likewise bullets 9–10 might gain traction if influential leaders can craft and advocate appropriate visions. A recent exemplar is the partnership announced between The Gates Foundation and OpenAI to advance AI capabilities for health in Africa with a goal to impact 1,000 primary healthcare clinics by 2028 (109). In advancing these opportunities, due consideration will also need to be given to aspects of security, privacy and medical ethics. For example, appropriate standards will need to be identified with regard to informed consent criteria, the ethics and privacy considerations of surveillance methodologies, and ensuring the provenance of any algorithms utilizing the data can be validated to minimize the potential for manipulation. Such oversight, ideally addressed by a pan-national working group could help ensure that any and all processes introduced are designed to avoid the exploitation of vulnerable groups, and adequate provision for future autonomy of the consenting patients is inbuilt.

## Conclusion and next steps

There are multiple opportunities to increase the heterogeneity of recorded health data in order to allow the benefits of AI technology to be fully capitalized upon. Likewise, broadening inclusivity in clinical trials and health decision making is of global significance and in societies interest to address. The observations and tactics outlined herein are presented in order to stimulate dialog among healthcare leaders, policy developers and providers, so that the goals can begin to be systematically addressed. Given the magnitude of the challenge globally it is perhaps unrealistic that immediate progress can be made on all opportunities presented in this review. However, we are confident that the breath of available options is wide enough to allow multiple approaches to develop in the near term. The purpose of outlining the entirety of the challenge in this forum is to allow interested parties to see how their contributions relate to other initiatives and opportunities within the overall framework, and to consider alliances and partnerships to advance otherwise isolated efforts.

The future holds much promise and there is reason for optimism in pursuit of these goals. It should be noted that digital literacy is improving dramatically in school curricula and the emerging generations of patients will likely be fully comfortable exercising transaction level approaches to their health data (110). Additionally, historical consumer concerns regarding security and privacy with digital approaches to banking have now been largely resolved with a majority of people having experience with one or more FinTech app. The lessons learned in developing trust in such technology based solutions to highly personal information can be instrumental in the capture of health related data and we encourage active dialog between the stakeholders from these industries (111). Likewise as new health related technologies emerge, the potential to leverage AI and datasets to amplify its utility is present and we encourage active dialog with nascent technology developers (112). The intent of this manuscript was to stimulate such discussions, and we look forward to witnessing many great developments over the years ahead.
